# Learning through multiple lenses: analysis of self, peer, nearpeer, and faculty assessments of a clinical history-taking task in Australia

**DOI:** 10.3352/jeehp.2018.15.22

**Published:** 2018-09-18

**Authors:** Kylie Fitzgerald, Brett Vaughan

**Affiliations:** 1 College of Health and Biomedicine, Victoria University, Melbourne, Australia; 2 Department of Medical Education, Melbourne Medical School, University of Melbourne, Melbourne, Australia; Hallym University, Korea

**Keywords:** Peer review, Self-assessment, Feedback, Educational measurement, Osteopathic medicine

## Abstract

**Purpose:**

Peer assessment provides a framework for developing expected skills and receiving feedback appropriate to the learner’s level. Near-peer (NP) assessment may elevate expectations and motivate learning. Feedback from peers and NPs may be a sustainable way to enhance student assessment feedback. This study analysed relationships among self, peer, NP, and faculty marking of an assessment and students’ attitudes towards marking by those various groups.

**Methods:**

A cross-sectional study design was used. Year 2 osteopathy students (n= 86) were invited to perform self and peer assessments of a clinical history-taking and communication skills assessment. NPs and faculty also marked the assessment. Year 2 students also completed a questionnaire on their attitudes to peer/NP marking. Descriptive statistics and the Spearman rho coefficient were used to evaluate relationships across marker groups.

**Results:**

Year 2 students (n= 9), NPs (n= 3), and faculty (n= 5) were recruited. Correlations between self and peer (r= 0.38) and self and faculty (r= 0.43) marks were moderate. A weak correlation was observed between self and NP marks (r= 0.25). Perceptions of peer and NP marking varied, with over half of the cohort suggesting that peer or NP assessments should not contribute to their grade.

**Conclusion:**

Framing peer and NP assessment as another feedback source may offer a sustainable method for enhancing feedback without overloading faculty resources. Multiple sources of feedback may assist in developing assessment literacy and calibrating students’ self-assessment capability. The small number of students recruited suggests some acceptability of peer and NP assessment; however, further work is required to increase its acceptability.

## Introduction

Health professional learners not only need clinical knowledge and skills, but also must learn to access, analyse, and apply new information. Developing the capability to evaluate their own performance enables learners to respond effectively to challenges in their current or future practice. Assessment and feedback also play an important role in developing both learner knowledge and evaluative judgement.

Evaluative judgement has been defined as “the ability to critically assess a performance in relation to a predefined but not necessarily explicit standard, which entails a complex process of reflection. It has an internal application, in the form of self-evaluation, and an external application, in making decisions about the quality of others’ work” (p. 661) [1]. For learners to develop evaluative judgement, they need to be assisted in developing an understanding of assessments and feedback literacy. Deeley and Bovill [2] suggested that assessment literacy involves learners becoming more knowledgeable in the language not only of their discipline of study, but of the assessments. Feedback literacy means that students comprehend what feedback is and how they can manage it, coupled with both the competency and attitude to obtain value from the feedback, while understanding the roles of students and teachers that contribute to these processes [3].

Assessments and feedback are typically the mainstay of the academic teaching staff who are expected to be able to provide students with credible feedback on their assessments. Learners’ skills also need to be developed to allow them to identify where changes to their practice are required, and how to undertake those changes. To facilitate the transition from learner to evaluator, alternatives to traditional faculty-marked assessments should be considered.

Ideally, health professional curricula should be underpinned by the principles of student-centred and self-directed learning, with assessment tasks designed to enable learners to reflect on and improve their performance. Feedback on these tasks should be obtained from multiple sources, as single-source feedback may not always capture all aspects of learners’ performance on an assessment. Self-assessment, peer assessment, and near-peer (NP) assessment may provide these sources [4]. Calibration of self-assessment may be facilitated through feedback from multiple sources and may also help develop learners’ evaluative judgement capability.

Peer assessment can be described as a process where students evaluate or are evaluated by their peers, consistent with the goals of selfdirected and collaborative learning [5]. Peer assessment may also help students build collaborative relationships and facilitate supportive reflections about their strengths and weaknesses. NP teaching is a valuable addition to student learning across a range of subject areas in health professions education [6]. NP teaching may be defined as instruction delivered by a more senior learner who is not a qualified professional. The benefits of NP teaching are thought to be related to the concepts of cognitive congruence and social congruence [6,7].

A body of health professions education literature suggests that peer assessments have value in conjunction with faculty grading and feedback for both academic and workplace learning and assessments [1,4,8]. Research has also explored the relationship between the marks provided by each of these groups. Studies have suggested that the correlation between peer and faculty marks is in the range of 0.29 to 0.69 [9-11], and peer assessment appears to be more closely aligned with faculty marking than self-assessment [11,12]. The literature with respect to NP assessment is lacking, however, and no studies have incorporated grades assigned from all 4 perspectives. The current study explored the relationships among self, peer, NP, and faculty assessments of students’ history-taking and communication skills using a simulated peer patient. Attitudes towards the extent to which peer and NP marks should contribute to the overall assessment grade were also explored.

## Methods

### Ethics statement

The study was approved by the Victoria University Human Research Ethics Committee (HRE17-178). Written informed consent was obtained.

### Study design

A cross-sectional study design was utilized. Year 2 osteopathy students enrolled at Victoria University completed an assessment on history-taking skills during a simulated patient scenario and the students conducted a self-reflection on their performance, a task that they had been exposed to in the first year of their training, from February 2016 to November 2016.

### Materials and subjects

Three participant groups were recruited: (1) group 1: year 2 bachelor of science (osteopathy) students (n= 86 were eligible to participate); (2) group 2: NP instructors (n= 4); and (3) group 3: faculty instructors (n= 14). The data collected included: (1) each learner’s video and written assessment task response; (2) short demographic survey for each of the NP (senior student teaching assistant) and faculty assessors; and (3) assessment score and completed feedback from self (the learner), a peer (a year 2 osteopathy student), an NP (a senior student teaching assistant), and a faculty member.

### Technical information

The assessment task involved a designated pair of students working together. One student was the practitioner and completed the clinical history while the other student acted as a simulated patient using a planned simulated case scenario. Students were instructed not to share the scenario with their peer beforehand. Students had undertaken training in portraying simulated patients prior to this assessment. Students made a video of less than 10 minutes’ duration that recorded the interaction. Students swapped roles, with the second student encountering a new case.

Each student reviewed his or her video using a rubric (Supplement 1) incorporating the SHARP debriefing tool [13]. The SHARP tool encourages students to identify aspects that they performed well, areas requiring improvement, whether the learning objectives for the task were met, and to outline a short plan for addressing the areas requiring improvement [13]. Students then uploaded the video and written assignment to the university learning management system for grading.

The researchers downloaded the submissions from students who agreed to participate. A randomization program allocated each student participant to a peer, NP, and faculty assessor. Submissions were emailed to each assessor with a 1-week deadline to complete the marking. Assessors received a short instructional video to facilitate assessment marking. Assessors used the same rubric (Supplement 1) for grading and providing feedback on the assessment task.

The year 2 student attitudes survey was adapted from Wen and Tsai [14] (Table 2). Some items were removed, including a section on online learning that was not relevant to this study. The modified University Student Peer/Near Peer Assessment questionnaire contained 14 items evaluated on a 5-point Likert-type scale (strongly disagree [1] to strongly agree [5]). This survey was also adapted to assess learners’ attitudes towards NP assessors. The modified survey included all previous statements, with ‘near-peer’ replacing the term ‘peer.’ Two additional items explored students’ opinions about the proportion of the grade that the peer (or NP) assessment score should contribute to (0%–100%) and whether they had any previous experience of peer (or NP) assessment (yes/no). The surveys were hosted in Qualtrics and a link was emailed to the student participants.

### Statistics

Data were entered and analysed via IBM SPSS ver. 24.0 (IBM Corp., Armonk, NY, USA). Descriptive statistics were generated for age, gender, and level of education, as well as for each NP and peer survey item. The relationship between different marker groups was assessed via the Spearman rho coefficient. Inferential statistics were used to ascertain any differences in perceptions by gender, and correlation statistics were generated for perceptions and age. Non-parametric effect sizes (r) were calculated where appropriate.

## Results

### Relationships among self, peer, near-peer, and faculty assessment

Year 2 students (n= 9), NPs (n= 3), and faculty (n= 5) were recruited for the assessment-marking component of this study. The participants in this component of the study were predominantly female (78%), with 78% aged between 18 and 26 years of age. Twenty-two percent of the participants had previous experiences with peer assessment.

**Fig. 1.**
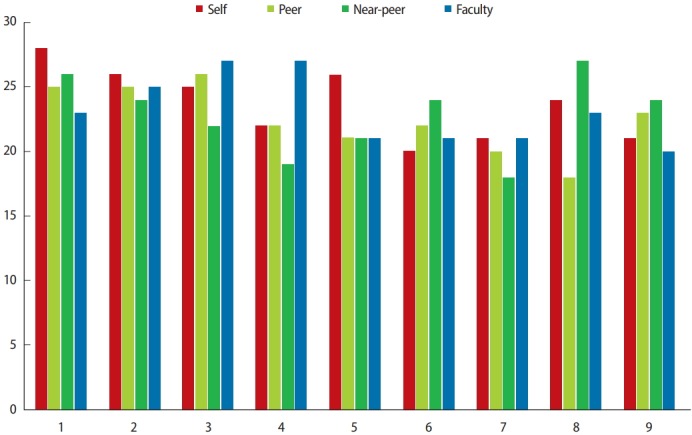
Assessment scores for each participant from each assessor group.

The mean final assessment scores for each group were: self, 23.61 (standard deviation [SD]= 2.69); peer, 22.39 (SD= 2.71), NP, 22.78 (SD= 3), and faculty, 23.11 (SD= 2.66) (Fig. 1). There was a moderate positive correlation between self and peer marks and between self and faculty marks (Table 1). A weak positive correlation was observed between self and NP marks (Table 1).

### Attitudes towards peer and near-peer marking

The Modified University Student Peer (or NP) Assessment questionnaire was provided to the group 1 cohort (Table 2). Seventy-two (n= 72) student participants completed the questionnaire (86% response rate), of whom 54.4% (n= 38) were female. The Cronbach alpha was 0.77 for NPs and 0.73 for peers.

Perceptions of peer and NP marking varied (Table 2) and where significant differences between peer and NP assessment were identified, these supported NP assessment. Male students were more likely to agree with the statements that “NP assessment motivates me to learn” (P= 0.01, r= 0.65), “Peer assessment motivates me to learn” (P= 0.004, r= 0.74) and “NP assessment helps me develop a sense of participation” (P= 0.008, r= 0.66), all with large effect sizes. Correlations between items and age were low for NP assessment (rho< 0.30) and trivial for peer assessment (rho< 0.17). Perceptions of NP or peer assessment were not significantly different between those with and without experience with either assessment approach.

**Fig. 2.**
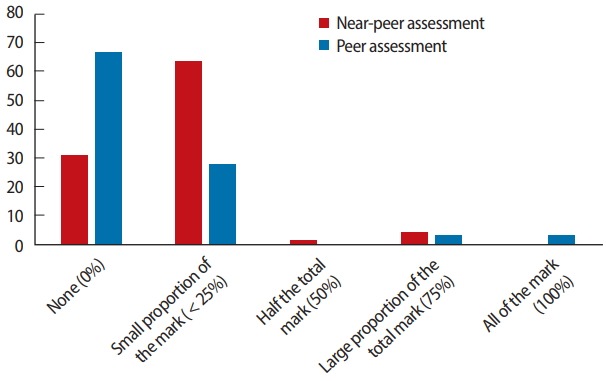
Student perception of contribution to their mark from a peer near peer.

Fig. 2 presents the percentages of the total grade that students perceived as appropriate for both NP and peer assessment. Sixty-seven percent of the participants suggested that peer assessments should not contribute to grades at all, while 63% suggested that NP assessments should contribute to up to 25% of a grade. The raw data are available in Supplement 2.

## Discussion

This study explored relationships among markers of an assessment and students’ perceptions of multiple sources of marking for a single assessment. Self and peer assessment grades correlated moderately with faculty grades, suggesting that there was a shared understanding of the assessment standard for the task. This finding is consistent with the literature identifying positive correlations between faculty and peer grading in medicine [1,11], providing support for this notion of shared understanding. This assertion is supported by the weak correlation between the peer and faculty marks when compared with the NP marks. The NP markers had not completed the same assessment task during their studies; however, they undertook training to mark the current assessment task. The shared understanding and experience of the assessment task appeared to be valuable, supporting the need for training sessions in which all marker groups are in one room at the same time. This shared understanding may have also emerged through the known overestimation of self-assessment grades in standardized patient tasks [15]. Furthermore, the highest mean group value for the task was demonstrated in the selfassessment group, consistent with other studies [12]. This may also be a reflection of the students’ higher self-efficacy [11], but this possibility requires exploration.

This study also explored students’ perceptions of both NP and peer assessment. Perceptions of peer and NP marking varied, with NP assessment favoured over peer assessment. The majority of the participants agreed that NP assessment could contribute to up to 25% of a grade, but that peer assessment should not contribute to grades. Grades for an assessment appear to be a factor contributing to the acceptability of a particular group contributing to the total mark for an assessment. This is positive with respect to self-assessment, as students should be able to trust in, and fine-tune, their selfassessment capacity, using the faculty marks to calibrate their thinking. It would be valuable to explore changes in self-assessment capacity over time [11] and across different assessments. Support for this assertion, and the use of peer assessment more broadly, is provided by the largely similar correlation coefficient between the faculty and peer marks [10]. Although the relationship between the sets of marks was acceptably close, students perceived that peer marks should not contribute to the total mark for the assessment. Further work to unpack participants’ apprehensions would be useful and could inform future research.

Students reported that peer assessments should not contribute to their grade for the assessed task, despite the training offered. Work by van Zundert et al. [5] has suggested that training positively influences student attitudes towards peer assessment, although that review was not focused on health students. Student participants in the current study agreed their peers offered value with respect to learning and skill improvements, and increased their sense of participation and interaction with peers. Where significant differences between peer and NP assessment were identified, these were in favour of NP assessment, with medium to large effect sizes. Students perceived NP assessment to be a fair way to assess the task and also to provide a small contribution to the overall mark for the assessment.

The involvement of NP assessors in assessment tasks appears to have some value, and students agreed that the NP mark could contribute a small percentage to the overall grade. It may be that participating as a NP assessor offers them a chance to foster their assessment and feedback literacy [7]. NP assessment may also provide a sustainable approach to assessing learner work, benefitting all stakeholders.

Interestingly, males were more likely to perceive NP assessment as motivational, with a positive influence on their sense of participation. These differences demonstrated large effect sizes, suggesting that some unidentified factors may influence females’ less positive perceptions of the value of this approach. This possibility requires further investigation and is an interesting avenue for further research.

Although the literature suggests there is perceived value in peer assessment [5], this was only borne out to some extent in the current study. This result suggests that more work to highlight the value of peer assessment may be necessary.

### Limitations

This small-scale study had limitations with respect to sample size, self-selection bias, and the NPs not necessarily having carried out the same assessment task in their own training. Notwithstanding these limitations, peer, self, and faculty marking provide an opportunity to implement sustainable assessment practices. The issue of whether peer and self-assessment marks should contribute to a final grade requires further work, and explicitly addressing student assessment literacy may help to improve the acceptance of peer assessment.

### Conclusion

In conclusion, self and peer assessment grades from year 2 osteopathy students correlated moderately with faculty grades for a clinical history-taking task. Multiple sources of feedback may assist in developing assessment literacy and help calibrate a students’ self-assessment capability. Perceptions of peer and NP marking varied, with NP assessment favoured over peer assessment.

## Figures and Tables

**Table 1.** Correlations (rho) between marks from various assessors

Assessor	Self	Peer	Near-peer
Self	-	0.38	0.25
Peer	0.39	-	0.13
Near-peer	0.25	0.13	-
Faculty	0.43	0.41	-0.043

**Table 2.** Modified University Student Peer (or near-peer) Assessment Questionnaire

	Near peer assessment		Peer assessment		P-value (effect size)
	Mean ± SD	Median	Mean ± SD	Median
Peer (or near-peer) assessment is helpful to my learning	4.03 ± 0.65	4	3.81 ± 0.82	4	0.047 (0.27)
Peer (or near-peer) assessment makes me understand more about teacher’s requirement	3.69 ± 0.85	4	3.29 ± 0.90	3	< 0.01 (0.43)
Peer (or near-peer) assessment activities can improve my skills in verbal communication	3.99 ± 0.81	4	3.46 ± 0.81	4	0.3
Peer (or near-peer) assessment activities motivate me to learn	3.63 ± 0.77	4	3.46 ± 0.81	3	0.19
Peer (or near-peer) assessment activities increase the interaction between my teacher and me	3.43 ± 0.97	3	2.97 ± 0.90	3	< 0.01 (0.47)
Peer (or near-peer) assessment helps me develop a sense of participation	3.75 ± 0.77	4	3.64 ± 0.76	4	0.41
Peer (or near-peer) assessment activities increase the interaction between my classmates and me	3.93 ± 0.77	4	3.99 ± 0.79	4	0.47
I think peer (or near-peer) assessment is fair to assess students’ performance	3.61 ± 0.89	4	2.97 ± 1.03	3	< 0.01 (0.64)
Peer (or near-peer) assessment activities help me understand what other classmates think	3.99 ± 0.70	4	4.13 ± 0.68	4	0.20
The teacher should develop criteria of peer (or near-peer) assessment activities for students	3.65 ± 0.75	4	3.67 ± 0.83	4	0.87
Students should participate in the development of criteria for peer (or near-peer) assessment activities	3.60 ± 0.70	4	3.59 ± 0.91	4	0.90
I think students should not be responsible for marking assessments	2.68 ± 1.01	3	3.89 ± 1.08	4	< 0.01 (0.65)
Peer assessment is time-consuming			3.57 ± 0.81	4	
The marks I give to classmates are affected by the marks given to me			3.57 ± 0.81	3	3

Modified from Wen and Tsai. High Educ 2006;51:27-44 [14].

SD, standard deviation.

## Supplementary material

Supplement 1. Assessment rubric

Supplement 2. Data files are available from https://doi.org/10.7910/DVN/LXPAOL.

Supplement 3. Abstract recording.
