# Supplementary material for: Learning through multiple lenses: analysis of self, peer, nearpeer, and faculty assessments of a clinical history-taking task in Australia
Source: J Educ Eval Health Prof. 2018 Sep 18;15:22. doi: 10.3352/jeehp.2018.15.22 (PMC6249140; doi:10.3352/jeehp.2018.15.22)
Supplement: Supplementary file 1 — Supplement 1. Assessment rubric [file jeehp-15-22-suppl1.pdf]

Supplement 1. Assessment rubric

Task	Description	Learning outcome assessed	Word count guide	Total marks	Marking rubric: 2 Good	Marking rubric: 1 Fair	Marking rubric: 0 Poor
1	Students will provide a video file (mp4 or Quicktime) that demonstrates their health history taking skills as an osteopathic student practitioner with a peer acting as a simulated patient.	4.4.1	Video (maximum length 10 minutes)	2	Video is in required format (mp4 or QuickTime) and is 10 minutes or less in length. Video provided accurately demonstrates the simulated patient and practitioner roles.	Video is in a format other than the prescribed format (mp4 or QuickTime) or 10 minutes or more in length or video provided does not clearly demonstrate the simulated patient and practitioner roles.	Video is not submitted or video is not in an accessible format.

Task	Description	Learning outcome assessed	Word count guide	Total marks	Marking rubric: 4 Excellent	3 Very good	2 Good	1 Fair	0 Poor
2	Record the clinical history on a mock version of the electronic medical records system used in the VU Osteopathy Clinic	4.4.1.	Unrestricted	8	All details of the patient and presenting complaint are recorded accurately, thoroughly, concisely, and using suitable terminology and abbreviations.	All details of the patient and presenting complaint are recorded accurately and thoroughly, using suitable terminology and abbreviations.	Most details of the patient and presenting complaint are recorded accurately, using mostly suitable terminology and abbreviations.	Most details of the patient and presenting complaint are recorded with some use of suitable terminology.	Patient details are not recorded or significant errors or omissions. Details of the presenting complaint are inaccurate and incomplete. Terminology is unsuitable.
					All details of the medical history are recorded accurately, thoroughly, concisely, and using suitable terminology and abbreviations.	All details of the medical history are recorded accurately and thoroughly, using suitable terminology and abbreviations.	Most details of the medical history are recorded accurately, using mostly suitable terminology and abbreviations.	Most details of the medical history are recorded with some use of suitable terminology.	Details of the medical history are inaccurate and/or incomplete. Terminology is unsuitable.

Task	Description	Learning outcome assessed	Word count guide	Total possible marks for section	Marking rubric: 4 Excellent	3 Very good	2 Good	1 Fair	0 Poor
3	Reflection part 1: review their video, the learning objectives of the task, and analyse their performance as a health professional using the "How did it go?" part of SHARP debrief tool	4.4.1	100 words	4	Student thoroughly, accurately, and succinctly identifies the aspects of the consult that were performed well	Student accurately and succinctly identifies the aspects of the consult that were performed well	Student accurately identifies the aspects of the consult that were performed well.	Student identifies some of the aspects of the consult that were performed well	Student does not provide any analysis of the aspects of the consult that were performed well
4	Reflection part 2: review their video, the learning objectives of the task, and analyse their performance as a health professional using the "Address concerns–what did not go so well and why?" part of SHARP debrief tool.	4.4.1.	100 words	4	Student thoroughly, accurately, and succinctly identifies the aspects of the consult that require improvement.	Student accurately and succinctly identifies the aspects of the consult that require improvement.	Student accurately identifies the aspects of the consult that require improvement.	Student identifies some of the aspects of the consult that require improvement.	Student does not provide any analysis of the aspects of the consult that require improvement.

(*Continued to the next page*)

Supplement 1. Continued

Task	Description	Learning outcome assessed	Word count guide	Total possible marks for section	Marking rubric				
					4	3	2	1	0
					Excellent	Very good	Good	Fair	Poor
5	Reflection part 3: review their video, the learning objectives of the task, and reflect upon their performance as a health professional using "Review learning points"<br>• Were your learning objectives met for this case?<br>• What did you learn about your history taking (communication, open/closed questions, active listening) skills?	4.4.1	150 words	4	Student identifies whether the learning outcomes were met and critically reflects on their history taking skill	Student identifies whether the learning outcomes were met and concisely reflects on their history taking skill	Student identifies whether the learning outcomes were met and provides some reflection on their history taking skill.	Student does not identify clearly whether the learning outcomes were met or the reflection is limited to a description of their history taking.	Student does not provide any reflection of their performance.
6	Reflection part 4: based on reflections 1, 2, and 3, provide a summary of action points to improve performance as a health professional using the "Plan ahead–what actions can you take to improve your future practice?" part of SHARP debrief tool.	4.4.1.	150 words	4	Student provides a succinct and detailed action plan that accurately addresses their observations of areas of strength & weakness, and provides detailed strategies for addressing these concerns.	Student provides an action plan that addresses all of their observations of areas of strength & weakness, and provides clear strategies for addressing these concerns.	Student provides an action plan that addresses most of their observations of areas of strength & weakness, and details some strategies for addressing these concerns.	Student provides an action plan that addresses some of their observations of areas of strength & weakness or provides limited strategies for addressing these concerns.	Student's action plan is incomplete or not provided.
7	Spelling and grammar			4	Spelling and grammar are entirely accurate. Writing is concise and resembles academic writing.	Spelling and grammar are mostly accurate. Writing is concise and resembles academic writing.	Spelling and grammar are often accurate. Writing is concise and partly resembles academic writing.	Spelling and grammar are somewhat accurate. Writing is concise and weakly resembles academic writing.	Spelling and grammar are largely inaccurate. Writing does not resemble academic writing.
Word count (reflection piece)		Subtract 5% of total mark if word count is > 550 words.							
Final score for HBS2404 = / 30 marks									
